# Stability of Metabolic Correlations under Changing Environmental Conditions in *Escherichia coli* – A Systems Approach

**DOI:** 10.1371/journal.pone.0007441

**Published:** 2009-10-15

**Authors:** Jedrzej Szymanski, Szymon Jozefczuk, Zoran Nikoloski, Joachim Selbig, Victoria Nikiforova, Gareth Catchpole, Lothar Willmitzer

**Affiliations:** 1 Max-Planck Institute for Molecular Plant Physiology, Potsdam, Germany; 2 Institute of Biochemistry and Biology, University of Potsdam, Potsdam, Germany; 3 Timiryazev Institute of Plant Physiology, Russian Academy of Sciences, Moscow, Russia; Auburn University, United States of America

## Abstract

**Background:**

Biological systems adapt to changing environments by reorganizing their cellular and physiological program with metabolites representing one important response level. Different stresses lead to both conserved and specific responses on the metabolite level which should be reflected in the underlying metabolic network.

**Methodology/Principal Findings:**

Starting from experimental data obtained by a GC-MS based high-throughput metabolic profiling technology we here develop an approach that: (1) extracts network representations from metabolic condition-dependent data by using pairwise correlations, (2) determines the sets of stable and condition-dependent correlations based on a combination of statistical significance and homogeneity tests, and (3) can identify metabolites related to the stress response, which goes beyond simple observations about the changes of metabolic concentrations. The approach was tested with *Escherichia coli* as a model organism observed under four different environmental stress conditions (cold stress, heat stress, oxidative stress, lactose diauxie) and control unperturbed conditions. By constructing the stable network component, which displays a scale free topology and small-world characteristics, we demonstrated that: (1) metabolite hubs in this reconstructed correlation networks are significantly enriched for those contained in biochemical networks such as EcoCyc, (2) particular components of the stable network are enriched for functionally related biochemical pathways, and (3) independently of the response scale, based on their importance in the reorganization of the correlation network a set of metabolites can be identified which represent hypothetical candidates for adjusting to a stress-specific response.

**Conclusions/Significance:**

Network-based tools allowed the identification of stress-dependent and general metabolic correlation networks. This correlation-network-based approach does not rely on major changes in concentration to identify metabolites important for stress adaptation, but rather on the changes in network properties with respect to metabolites. This should represent a useful complementary technique in addition to more classical approaches.

## Introduction

Biological systems exposed to new environmental cues react by adapting their cellular and physiological program in order to optimize the likelihood of survival under the new condition. Due to the crucial role of adaptation, there have been numerous studies investigating the response of biological systems to environmental challenges [1], [2], [3]. The relative biological (physiological) simplicity of unicellular organisms (*e.g., Saccharomyces cerevisiae, E. coli*) and availability of their respective annotated genomes have prompted the employment of integrated, systems-oriented approaches, often relying on transcriptome data, to study the response towards changing environment [3], [4]. Examples comprise oxidative stress [5], [6], [7], temperature shifts [8], [9], [10], [11], [12], [13], or changes in carbon sources, such as the classical lactose diauxie [14], [15].

In contrast to the abundance of systems-oriented approaches describing changes on the transcriptome or interactome level, relatively few studies have employed the metabolome [16], [17]. Metabolite concentrations represent a vital readout of the cellular machinery which may even be considered to be closer to the complex phenotype than transcripts or proteins [18], [19], [20].

Based on an established high-throughput metabolic profiling technology [21], [22], we conducted a comprehensive study focusing on the dynamic response of *E. coli* to four different environmental perturbations: cold, heat, oxidative stress, and a lactose diauxic shift, on the metabolite level. The resulting data are explored via the reconstruction and interpretation of networks, based on metabolite-metabolite pair wise correlation [17], [23]. The reconstruction of such a network, which presents a binary simplification of the underlying covariance matrix for a certain significance threshold, renders it possible to apply network analysis tools, thereby bringing a new context of data analysis and introducing a system-wide level of reasoning. Network-based analysis of metabolite correlations should be regarded as an abstraction bridging the gap between purely topological approaches [24] and flux-balance-constrained methods (*e.g.*, Flux Balance Analysis (FBA) [25] and Metabolic Control Analysis (MCA) (for a review see, [26]).

A direct relationship between metabolic pathways, biological function, and metabolite correlations is not yet well-established [27]. Recent approaches have demonstrated that the covariance matrix in its classical form does not provide enough information to reverse engineer an underlying enzymatic system [28]. Nevertheless, it has been concurrently shown not only that the covariance matrix can represent a characteristic fingerprint for a physiological state of an investigated system, but also that it changes with steady-state concentrations of metabolites in mutant organisms [29] and reflects such complex processes such as an increase in anaerobic respiration in hepatic tumor [30]. Thus it is reasonable to assume that both differences and commonalities between any measured systems could be reflected in the underlying features of such a reconstructed network.

As outlined above we analyzed the metabolic response of *E. coli* towards four different stresses and control growth conditions. One of the goals was to identify candidate metabolites which are important for the organism to adjust to the new environmental challenge. Classically these metabolites are identified by either mutant screens or looking for metabolites which display a major change in concentration. (e.g. Brauer et al. 2006 [31]). As we show in following sections, the analysis of the metabolite-metabolite covariance matrix is a new approach to detect such metabolites. In addition it provides a platform to interpret the observed changes on the systemic level.

In the following sections we will address the following questions: (1) the stability of the correlations for different environmental conditions; (2) the relationship between metabolite-metabolite correlations with respect to existing knowledge represented in pathway databases (*e.g.*, EcoCyc); (3); the extent to which the properties of the reconstructed networks reflect the biological differences resulting from the studied environmental cues and, finally, (4) the role of particular metabolite in the conditional correlation network rewiring.

## Results and Discussion

### Early and Mid-Log Phase During Control Growth Display the Smallest Number of Metabolic Changes

**Figure 1:**
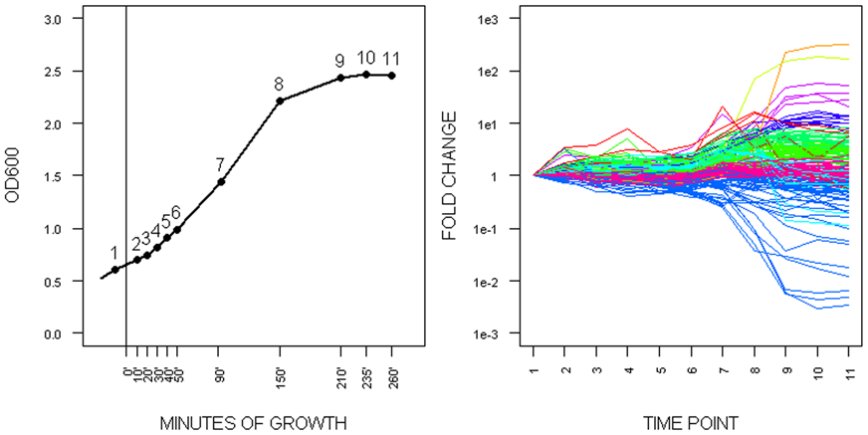
Metabolic changes during *E. coli* culture growth. (A) Growth curve (optical density) of unperturbed *E. coli* culture. Numbers of respective sampling time points are marked in the curve. Time point 0 minutes marks the application of the respective stress condition. (B) Relative changes of metabolites pools normalized to culture OD and time point 1. Fold change is presented on log_10_ scale. To reveal main trends of metabolic changes 10 K means clusters are color coded.

As outlined in the Introduction we are interested to analyze the response of *E. coli* when exposed to four new environmental conditions. A prerequisite for such an analysis is the identification of a growth phase where metabolite changes are minimal thus allowing to assign changes in metabolites to the applied stress condition and not to changes in the general growth condition. To identify this time window we in a first pilot experiment followed the metabolic changes *E. coli* displays during a normal batch-type growth curve in an unperturbed state ( cf. fig. 1a) (cf. Materials and Methods for the exact conditions). The results of this analysis are shown in Figure 1b in the form of a k-means clustering using an arbitrary k-value of 10. It shows that the metabolic complement (at least as assayable via our analysis) remains relatively constant during early and mid logarithmic growth phase ( time points 1–6 in Figure 1b) which is not surprising and in agreement with transcriptomic data [32]. In striking contrast massive changes in metabolites occur at later growth phases specifically upon entry into stationary phase (cf. Figure 1a, b; time 8–11). Based on this observation we decided to apply the various stresses at the early logarithmic growth phase, equivalent to time point o minutes in Figure 1a and to follow the response of the cells over a total number of five time points during the adjustment phase (defined by the duration of the respective lag phase, cf. supplementary Figure S1 for an example). All experiments were performed in three independent biological replicates; moreover, for each time point and each biological replicate, three samples were taken, resulting in nine samples (for each time point). Obtained data were normalized to internal standards and optical density of the culture at the moment of sampling (*see* *Materials and Methods*). The sampling procedure, including 5 seconds of filtering on a 0.45 µm filter was chosen as being extensively used in microorganisms studies [33] and as shown to be superior in comparison with deep temperature quenching [34]. As it is crucial for covariance analysis, that measured metabolites are quantified precisely and in all samples, from a total pool of 247 metabolites identified in the analyzed samples 170 most reliable peaks were selected along the criteria of quality of chromatographic separation, confirmed biological source, detection in all analyzed chromatograms and a reasonable signal to noise ratio (see *Materials and Methods* for more details). Analysis of the data obtained for the different stress treatments by k-means clustering are shown in supplementary Figure S2 together with a quantification of the similarity between the different clusters using Mantel statistics.

### Metabolite Covariance Allows for Identification of Significant Correlations: Development of the Homogeneity Test

**Figure 2:**
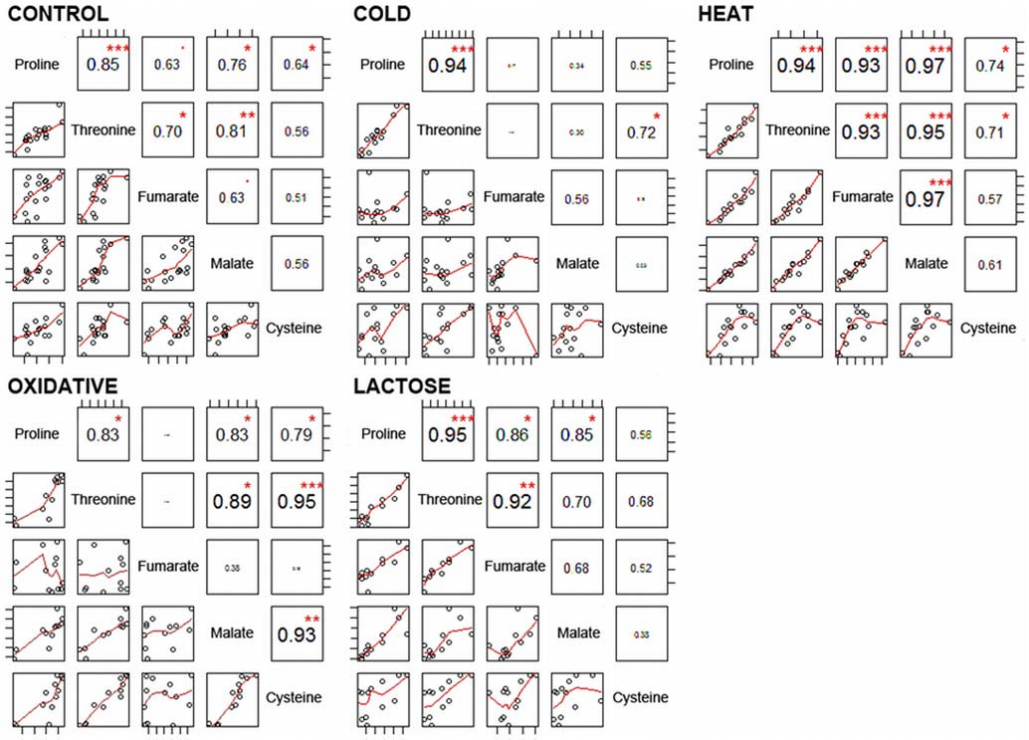
Example subset of metabolites showing different patterns of correlations across the treatments. Significant correlations are marked by red stars pinpointing values significant with p-value respectively: *** <10^−5^, ** <10^−4^, ** <10^−3^, ‘<10^−2^.

While the initial question is that of identifying whether or not the covariance displayed by the relative metabolite concentrations is sufficient to identify significant correlations, of additional interest is the issue of how these correlations are conserved among the applied conditions (treatments). Figure 2 shows an example matrix of metabolite-metabolite correlations for a small subset of metabolites and the different patterns exhibited following each treatment. As illustrated, the observed pair-wise correlations fall into one of three categories: those which correlate under all applied conditions (*e.g.*, proline-threonine), only under one or more (but not all) conditions (*e.g.*, fumarate-malate), and those which display no significant correlation under any of the conditions tested (*e.g.*, fumarate-cysteine).

It is expected that any such classification is greatly influenced by the applied significance threshold. Thus, for the cysteine-proline pair, following both cold and lactose stress, correlations are nevertheless apparent, despite falling below the significance threshold. A similar observation can be made for the malate-fumarate pair: although this correlation was found to exceed the highest significance threshold only in the case of heat, a correlation can also be identified by visual inspection in the case of lactose shift and under control conditions.

We are interested in both, stable (observed under all experimental conditions) and environment-specific correlations. A classical method of correlation network reconstruction applies an arbitrary significance threshold to the correlation coefficients [35]. When comparing several networks, on the same nodes (metabolites) but differing in their wiring, the simplest method to find a stable component is to determine their intersection. However, this may lead to a considerable loss of information, particularly when correlation coefficients in any of the treatments fall marginally short of the significance threshold (as exemplified in Fig. 2). A technique with lower information loss, used here, relies on the *homogeneity test* which can be employed to assess the correlations (and thus the resulting network) that can and should be regarded as stable or specific in addition to the significance threshold tests. To this end, we use a two step approach (detailed in *Materials and Methods*): (1) a union network is reconstructed where there is a link between nodes (metabolites) if and only if this particular pair of metabolites correlates significantly with a Bonferroni corrected p-value of <0.01 under at least one environmental cue (2) a homogeneity test is performed on the Z-scores of correlation coefficients from different treatments for each edge of the union network with a test p-value threshold of 0.01. This “soft intersection” method renders it possible to avoid a sharp correlation coefficient cutoff and makes the robustness of a particular correlation dependent on all compared conditions and not on each one, separately. A decision to qualify a given correlation as stable or stress-specific must thereby be supported by both the correlation significance test and the homogeneity test of the correlation coefficient's distribution in all experimental conditions.

### Reconstructed Networks Differ in the Number of Edges and Network Density

As a first step in our analysis we reconstructed a correlation matrix for each of the five conditions representing bootstrapped Pearson correlation coefficients between pairs of metabolites. (Cf. Supplementary Table S2 for all correlation values for all pairs of metabolites). Correlation networks were obtained by discretizing of these matrices along significance threshold criteria (see *Materials and Methods* for details); note that in this step the significance threshold is the only criterion applied (Table 1). Considering two related parameters, network density and edge number, reveals that the control and heat networks display the highest density and largest number of edges, and in doing so, along with the cold stress network, they also possess the smallest number of isolated nodes. Furthermore, the data suggest an (inverse) relationship between network density and average variance of the metabolite time courses. Thus heat stress and control conditions display the lowest average variance (being an average value from values of variances exhibited by all metabolites in response to particular stress) over all metabolites measured (0.226 in the case of control and 0.352 for heat stress) but the greatest network densities.

**Table 1:** Basic global properties of investigated networks.

	Control	cold stress	heat stress	oxidative stress	lactose shift	networks intersection	stable component
Number of edges	243	156	341	138	186	9	169
network density	0.029	0.024	0.042	0.012	0.016	0.0008	0.01512304
isolated nodes	47	44	42	69	55	132	63
Average variance	0.2267887	1.696756	0.3523608	0.6826608	0.4812		

Despite the considerable difference in network density, a substantial degree of network overlap (as determined by the number of significant edges common to the two conditions compared) is observed. This degree ranges from 6% common edges between the two most disparate stresses (oxidative and heat stress) to the 22% conserved edges observed when comparing cold and heat stresses (cf. Table 2). Even though not so striking in terms of percentage, this overlap is statistically highly significant as displayed by a comparison with an exemplary random network (p-values exceed 1e-100 when applying the Fisher exact test). Only 9 metabolite pairs reach the absolute significance threshold in all conditions, which may be regarded as unexpected in the case of a system constrained by the same underlying metabolic pathways. However, as we discuss later, this result is an underestimation caused by the strict “yes or no” discretization criterion, testing the correlation homogeneity between different conditions allows detection of much denser stable network components.

**Table 2:** Networks comparison.

		Control	Cold stress	Heat stress	Lactose shift	Oxidative stress	Randomized network	Stable component
Control	overlap percentage	-	12.3***	20.6***	13.6***	10.3***	1.6	29.6***
	intersecting edges	-	30	50	33	25	4	72
	path difference	-	1.11***	1.73***	1.19**	1.11***	1.26	0.80*
Cold stress	overlap percentage	19.2***	-	22.4***	14.7***	11.5***	1.3	29.5***
	intersecting edges	30	-	35	23	18	2	46
	path difference	1.11***	-	1.78**	1.36***	0.96***	1.49	0.92***
Heat stress	overlap percentage	14.7***	10.3***	-	9.4***	6.5***	1.8	26.7***
	intersecting edges	50	35	-	32	22	6	91
	path difference	1.73***	1.78**	-	1.50***	1.69***	1.99	1.16***
Lactose shift	overlap percentage	17.7***	12.4***	17.2***	-	8.1***	1.1	34.9***
	intersecting edges	33	23	32	-	15	2	65
	path difference	1.19**	1.36***	1.50***	-	0.98***	1.51	0.82***
Oxidative stress	overlap percentage	18.1***	13***	15.9***	10.9***	-	0	31.9***
	intersecting edges	25	18	22	15	-	0	44
	path difference	1.11***	0.96***	1.69***	0.98***	-	1.82	0.66**
random	overlap percentage	2.4	1.2	3.6	1.2	0	-	0.6
	intersecting edges	4	2	6	2	0	-	1
	path difference	1.26	1.49	1.99	1.51	1.82	-	1.25
Stable component	overlap percentage	42.6***	27.2***	53.8***	38.5***	26***	0.6	-
	intersecting edges	72	46	91	65	44	1	-
	path difference	0.80*	0.92***	1.16***	0.82***	0.66**	1.25	-

Percentage of the edge overlap, number of intersecting edges and difference in average path length between individual stress networks, stable component and randomized network. Difference in average path length describes how the shortest path between two corresponding metabolites differs in two different networks (see Materials and Methods for more details). Significant results are marked with stars (*<0.1,**<0.001,***<0.0001) highlighted with bold font (p-value <0.001). Numbers mean what percent of the row network is shared with a column network. A random network is only an example – the significance tests were done for 1000 randomization permutations.

The overlap between the individual networks and the stable component ranges from 26% to 53% (Table 2). This comparison may however, not be straightforwardly interpreted, since the stable component in defined experimental conditions permits correlations below the absolute significance threshold. We note that this is a direct consequence of the criterion used for including an edge in the stable component (which may not appear in the networks for separate conditions and would have been filtered out by the simple intersection method), which ultimately renders the method more powerful. Despite this apparent difference, the proportion of the stable component to the entire network is only varying by a factor of two (from 26% to 53%) indicating largely constant ratio between stress-specific network rewiring and the homeostasis of the system represented by its stably wired backbone. A difference in shortest path length between two metabolites in different networks is also, in most cases, significantly smaller than those obtained for random network rewiring (see *Materials and Methods*).

### Analysis of the Stable Network Component: Modular Structure and Scale-Free Topology

**Figure 3:**
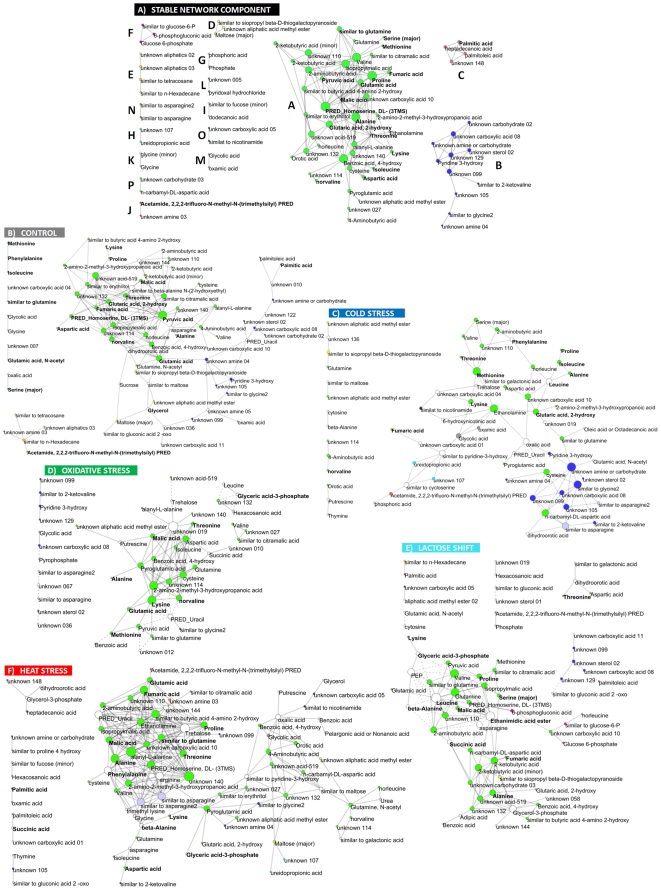
Metabolic correlation networks inferred in the analysis. (A) Structure of the stable network component composed of all metabolites exhibiting at least one stable correlation and correlations selected as stable using criteria described in text. Each component of the stable network is marked by different color. Size of the node is proportional to its degree. Metabolites exhibiting significant change in concentration in at least one of the applied conditions are marked with bold font face. All components and metabolites with known or approximate chemical structure are listed in table 3. (B–F) Stress specific networks composed of all connected metabolites and correlations specific only for particular condition. Positive correlations are represented by solid edges; negative correlations are represented by dashed edges. Components of the stable network are mapped by node colors. Size of the node is proportional to its stress specific degree. Metabolites exhibiting significant change in concentration in particular condition are marked with bold font face.

For construction of the stable component network, the correlation significance and homogeneity tests were applied to the complete data set, yielding 169 stable links (correlations) organized in 16 connected components, the largest of which containing 55 nodes (metabolites) and 15 smaller ones containing between two and six (Figure 3a, cf. also Table 3). From the total pool of metabolites, 63 remained isolated; however, only nine of these showed no significant correlation in any of the treatments. The number of isolated metabolites is stable and largely independent of the correlation significance criterion. Thus when the correlation significance criterion was used without correcting for multiple comparisons (p-value threshold fixed on 0.01), the number of isolated metabolites only slightly decreases down to 55. These findings validate the robustness of the method against varying threshold values. Therefore this observation indicates that connectivity of the metabolites in the stable network component is indeed related to their fluctuations as a result of prevailing environmental condition and not to statistical significance threshold.

**Table 3:** Components members of the stable network.

A	B	I
Malate	3-hydroxy-pyridine	dodecanoate
Fumarate	similar to 2-ketovaline	similar to fucose
Pyruvate	similar to glycine	J
4-hydroxy-benzoate	unknown carbohydrate	trifluoro-N-methyl-N-trimethylsilyl-acetamide
Ketobutyrate	unknown carboxylic acid	unknown amine
4-aminobutyrate	unknown amine or carbohydrate	K
Orotate	unknown sterol	Gly
Ethanolamine	unknown amine	L
similar to erythritol	C	pyridoxal-hydrochloride
similar to 4 -amino-2-hydroxy-butyrate	Heptadecanoate	M
Pyroglutamate	palmitoleic acid	Oxamate
2-hydroxy-glutarate	palmitic acid	Glycolate
2-amino-2-methyl-3-hydroxy-propanoate	D	N
Homoserine	similar to maltose	Asp
Norvaline	similar to D-thiogalactopyranoside	similar to Asp
Norleucine	E	O
Val	similar to tetracosane	similat to nicotinamide
Met	unknown aliphatics	unknown carboxylic acid
Cys	unknown aliphatics	P
Thr	F	unknown carbohydrate
Glu	6-phospho-gluconate	n-carbamyl-aspartate
Ala	glucose-6-phosphate	Q
Ile	similar to glucose-6-phosphate	lignoceric acid
Asn	G	Xylitol
Ser	Phosphate	2-hydroxybutyrate
Ala	H	stearic acid
Gln	Ureidopropionate	unknown carboxylic acid
Lys		unknown amine
Pro		

Only identified or partially identified metabolites are included in the table.

Analysis of the stable component topology was performed by applying a goodness-of-fit test for normal, power-law, and exponential distributions. Using a non-linear least squares method and based on the *p*-values obtained (<0.0001 in both cases) the latter two were deemed most probable. Subsequent calculation of the Akaike information criterion score [36], [37] indicated that the power-law is more accurate in the case of the stable component network (for k = −0.7588). The power-law degree distribution is widely referred as a salient property of hierarchical scale-free networks and has been reported to be characteristic not only for biological, but also for other self-organizing systems [38], [39], [40]. Moreover, these networks exhibit short average path length and hierarchical organization, marked by locally-dense globally-sparse architecture directly reflected in the large number of poorly connected nodes and a relatively low number of highly connected nodes, called hubs [41], [42]. This finding thus suggests the hierarchical organization of the stable component, where a relatively small fraction of the metabolites, the network hubs, is responsible for most of the observed network connectivity.

### Analysis of the Stable Network Component: Local Network Properties Are Related to Underlying Metabolic Pathways

Networks constructed as described in the preceding paragraph are based on experimental data in the form of metabolite measurements. In order to see whether or not they reflect existing knowledge we compared the connectivity of the hub metabolites in the stable network described above with their connectivity in the metabolic pathways as defined in the EcoCyc database [http://www.ecocyc.org] [43], [44],. To this end we reconstructed the *E. coli* metabolic network which was subsequently reduced for so called *currency metabolites* (*e.g.*, H2O, CO2, ATP and cofactors, see *Materials and Methods* for a complete list); these appear as major connectors in original networks, but as being only donors, acceptors or carriers for particular chemical group transfers they play rather a role of reaction parameters and do not constitute a defined step in metabolic pathway (discussed more deeply and applied in Fell and Wagner 2000 [45], Ma and Zeng 2003 [46] and Albert 2005 [47]). Among 150 measured metabolites, 68 were annotated with a name matching an entry in the EcoCyc database, of which 49 could be mapped directly to the major connected component of the metabolic pathways and thus used for subsequent analysis. A general shift of the connectivity value for this subset of metabolites was estimated by a nonparametric location shift test and a significant bias of all measurable metabolites in the direction of more connected ones in the metabolic pathways was detected (*p*-value of 5.408e-06 for alternative hypothesis).

In a second test we compared the pathway connectivity of hubs in the stable component network with that of metabolites having no hub property in the stable component network. This test clearly showed that a high connectivity in the experimental network is significantly related to the high connectivity in the metabolic pathways (location shift p-value <0.05). As the measured metabolites are scattered across the investigated pathways, local network properties (*e.g.*, connectivity) may have a negligible discriminatory power. Therefore, we also investigated local-global properties of the pathway network, specifically, the betweenness of measured metabolites, recently related to robustness of complex networks [48]. Also in this case, metabolites characterized by high betweenness in metabolic pathways are mostly those of high connectivity in the correlation network reconstructed from experiments (supplementary Table S1). As measured metabolites are rarely connected by a single reaction, shortest paths analysis, such as applied in betweenness, is a good candidate to bridge metabolite-metabolite correlations with metabolic pathways, especially since independence of local network connectivity was proven to be an intrinsic property of metabolic networks defining their robustness [49]. Thus in conclusion all three measures support the notion that the networks reconstructed based on the metabolic profiling data do reflect existing knowledge about biochemical pathways.

**Figure 4:**
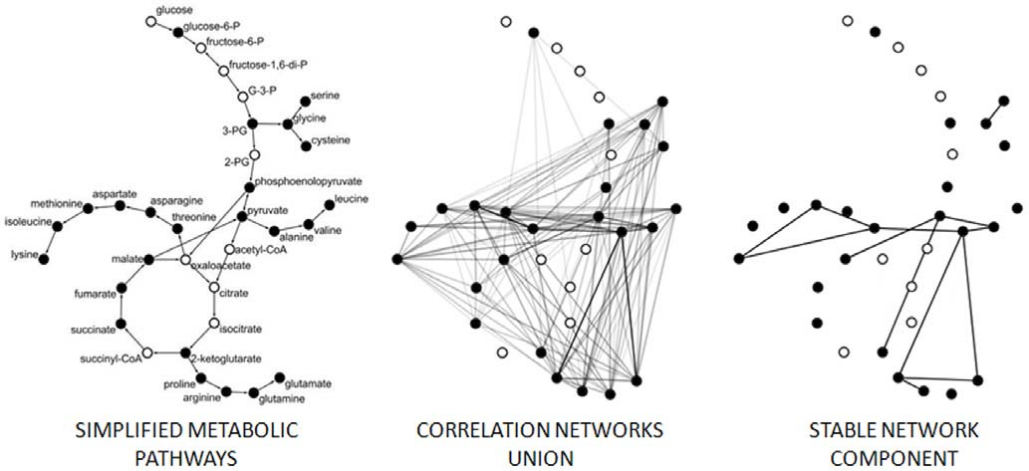
Overlap between correlation networks and metabolic pathways. (A) A selected subset of metabolic pathways network, including TCA cycle, glycolysis and synthesis of several amino acids. Metabolites measured in our experiments are marked black. (B) Union of correlation networks obtained in 5 investigated conditions. Edges are shaded according to the number of treatments in which they were found significant. (C) Stable network component identified by testing homogeneity of the correlations obtained in different conditions.

An exemplary comparison between a subnetwork of metabolic pathways and related system of stable correlations is shown on Fig. 4. It is important to note, that correlation between amino acids might be a result not only of their common biosynthetic pathways but also of their coordinated outflow for protein synthesis or conversely, a result of amino acids release as a result of protein degradation. Nevertheless we observe a significant and stable correlation between amino acids being or sharing common substrates, e.g. threonine/aspartate/lysine, proline/glutamate and proline/arginine, alanine/valine or glycine/serine. On the other hand correlations of the central metabolites such as pyruvate, succinate, malate or α-ketoglutarate might be more dependent on the biochemical pathways structure, but also of more unpredictable behavior because of their pleiotropic character. Even though, identification of correlations being stable across all studied conditions helps to detect close metabolic relationships, such as e.g. pyruvate/malate, pyruvate/α-ketoglutarate or pyruvate/valine.

### Analysis of the Stable Component Network: Identification of Functionally-Related Modules

Modularity of the stable component is directly represented by the presence of 16 disconnected components. No other hierarchical decomposition of the components gave a satisfactory result, suggesting that each of the disconnected components is relatively uniformly connected and may be treated as a structural network unit (Fig. 3a).

The major component A of the stable component network covers most primary metabolism and amino acids, representing a significant part of the homeostatic system. Component B consists mainly of unknown metabolites and as a result, functional characterization is unfortunately not possible. On the other hand, component C contains palmitic, palmitoleic and heptadecanoate acid and is therefore likely to be related to lipid metabolism. Cluster D contains maltose, and other sugars of unknown exact chemical configuration. Glucose-6-phosphate correlates tightly with gluconic acid-6-phosphate and some G-6-P related structure within component F. The isolated nodes are denoted as cluster Q (not shown in this figure but contained in the supplementary Table S1). These examples suggest that emerging correlations between metabolites, which are expected to be closely related by function, tend to also be stable.

To directly assess the relationship between correlation network structure and metabolic pathways, average shortest paths in the metabolic pathways were computed for the members of each network component and for each module. Use of shortest paths instead of the direct common links is advantageous because measured and annotated metabolites are rarely directly connected in metabolic pathways. The shortest path length, as minimal number of steps needed to reach one metabolite from another, allows working on a small subset of identified metabolites keeping in the same the information of the whole network structure. Additionally in order to correct for a potential bias introduced by the limited subset of metabolites annotated from our experimental system, the average EcoCyc shortest path distance for 1000 random sets of 49 metabolites was calculated. This resulted in an average value of 8.59, while performing the same analysis on the subset of 49 measured and annotated metabolites resulted in an average distance of 6.66. Thus we observe a significant location shift for the whole network (p-value <2.2e-16), indicating that the measured and annotated metabolites are in closer proximity on the metabolic pathways as compared to a subset of the same size chosen at random. Because of the low number of EcoCyc-mapped metabolites, only cluster A could be compared against isolated nodes, since all other clusters contain no or too few annotated metabolites. The result of this analysis leads to an average distance of 8.54 for unconnected metabolites as compared to 5.64 for metabolites of module A, thus significantly separating the two groups and arguing for structural constraints of the stable correlation network topology. Upon omission of the two most distant metabolites, orotate and 4-hydroxybenzoate, from module A, an even lower value of 4.7 is obtained. Thus, we suggest that metabolic pathways are extensively reflected in conserved metabolic correlations, although not exclusively in a trivial path length-dependent fashion. The noticeable occurrence of conserved correlations between metabolites, displaying a large distance in the metabolic pathways, strongly suggests more complex interactions, hinting at allosteric regulatory mechanisms. We point out that similar analysis of condition-specific networks, treated separately, does not show comparable results.

With regard to these findings, the reliance of our approach on time-course data offers a more powerful way for understanding the dependencies in a biological system arising as a result of biochemical reactions. While similar results can be obtained from studies reliant on biological variability, they are more difficult to interpret [50]. Finally, our results suggest that only a limited number of these correlations, remaining stable across the different conditions, may carry additional information about underlying biochemical reactions, yet to be assessed

### Stress-Specific Networks: Hypothesis Creation for Identification of Candidate Metabolites Important for Condition-Specific Response

Having considered the stable network, next we focus the analysis on stress-specific network components (Fig. 3b–f).

To select the stress-dependent correlations, the previously described stable network component was used. This is, of course, reliant on the tenet that a stress-dependent network may be defined by interactions which appear significant following a particular stress, but are not present in the stable component. Hence, a desired network can be obtained by simply subtracting one from the other (Table 2). From these networks, we concluded that heat and cold stresses share the highest percentage of stress-related connections, which makes this pairing suitable for determining common responses.

The other remaining question concerns those correlations which appear upon each of the applied stresses, but which are not present in control conditions. In order to extract them using we obtained a “soft intersection” of 4 stress networks and discarded those correlations which are either significant or close to significance in control conditions. The result is the identification of three classes of stress-specific correlations: firstly, those which are specific for all stresses and can therefore distinguish stress conditions from control; secondly, those which are specific for both cold and heat stress; and thirdly, correlations specific for only one particular treatment (Table 4). The number of correlations which are significant for only a single condition is surprisingly large; in all cases except cold stress about 50% of all correlations are stress-specific (compared to Table 1).

**Table 4:** Global properties of the condition-specific networks.

	Control specific	cold stress specific	heat stress specific	oxidative stress specific	lactose shift specific	heat and cold stress intersection	Stable component of stress conditions
number of edges	130	86	221	64	85	11	63
Network density	0.011	0.0076	0.019	0.0057	0.0076	0.0009	0.0056
number of orphans	77	83	61	112	94	140	102

Edge number, density and number of isolates in condition-specific networks.

From the available pool of links, only 63 links (correlations) form a network present in the four stress conditions but not in the control. This network is composed of four major disconnected components in which malate, Val, Ala, Pro, homoserine, 2-aminobutyrate and 3 unknown metabolites accumulate most of the network connectivity. 11 correlations were identified as specific to both heat and cold, with ethanolamine, Thr, Phe, Pro, 2-aminobutyrate, 2-amino-2-methyl-3-hydroxypropanoate, uracil, and 2 unknowns as the only connected metabolites (data available in supplementary Table S1, networks not shown).

To determine compounds whose abundance significantly changes in response to an applied condition, the time courses of individual metabolites were first studied to reveal those which significantly either accumulate or dwindle. A list of the most “responsive” metabolites was then generated for each treatment in turn (supplementary Table S1, mapped by bold font face on Fig. 3). The number of common and stress-specific connections in correlation networks was then compared between “responsive” and “non-responsive” metabolites and this then expressed as a ratio (table 5).

**Table 5:** Comparison of condition-specific and stable connectivity of the metabolites.

	Metabolite degree for single stress and combined networks		Metabolite degree when common connections are subtracted (only stress specific connections)	
Condition	Ratio	Test significance	Ratio	Test significance
Control	0.93	0.673	1.73	0.005
Cold stress	0.99	0.659	1.91	0.011
Heat stress	0.84	0.440	1.90	0.041
Lactose shift	1.73	0.163	4.19	2.85e-07
Oxdidative stress	1.71	0.071	4.67	8.92e-06
Temperature stress	1.73	0.131	1.87	0.125
Stable component	2.46	0.036	-	-

Degree of the most stress responsive metabolites is compared to the degree of non responsive ones and displayed as ratio. This comparison was done for single stresses including control and combined networks and for the same networks but subtracted for presence of the common correlations. The significance of the difference (i.e. degree of responsive vs non-responsive metabolites) is checked by Wilcoxon rank sum test.

The first notable observation concerns the extent to which large changes in a single metabolite results in a change of the network structure, expressed by the appearance of new correlations. We determined that most stress-responsive metabolites are significantly enriched for connections in the stable component, having on average, 2.46 times higher connectivity than non-responsive metabolites. This implies that the metabolites exhibiting the highest number of stable correlations are also those which also most radically respond to changes of environmental conditions. Alternatively, the first column of Table 5 suggests that the total connectivity of metabolites in any individual network is much less related to their response. This relationship becomes more significant only when stress-specific connections are isolated through the removal of those which are shared with the stable component. As a result, a large change in the concentration of a given metabolite does not necessarily induce a complete change of its environment in the network, although it appears to be tightly connected with the gain of new correlative neighbors. We believe this to be an important observation as it gives a strong rationale for the search of the new stress-related biomarkers via network analysis. Moreover, it also points out that the hub property could be indicative for the involvement of the metabolite in stress adaptation.

It is important to mention, that most of observed correlations are positive and only these seem to be conserved across different conditions. Significant negative correlations are only observed in case of three conditions, control, oxidative stress and the lactose shift. In each case however they contribute less than 15% of all significant correlations found. This disproportion between the number of negative and positive correlations is a common feature of correlation networks and has also been observed e.g. in case of transcriptome data and other highly clustered datasets [51], [52]. The reason for the overrepresentation of positive correlations must remain speculative, however it is self-explanatory that clusters of commonly co-regulated genes or metabolites will enrich the pool of positive correlations independently of general increase or decrease. Negative correlation between metabolites on the other hand is sometimes referred to direct or indirect substrate/product relationships respectively competing pathway branching [30]. This feature is met with a higher likelihood under conditions of limited nutrient resources, where pathways are competing for intermediates and the production of one metabolite requests a decreased pool of another one. In this respect it is interesting to note that the diauxic shift experiment where cells are experiencing a shortage in glucose supply displays the highest proportion of negative correlations (25 out of 186 significant correlations) which is in vast excess of the control condition ( 7/243 negative versus total correlations) but similar to the oxidative stress experiment ( 19/138).

### Network-Based Analysis Leads to the Identification of Numerous Metabolites of Hypothetical Importance for Stress Adaptation

As shown in Fig. 3a–f, all stress-based networks exhibit a high number of specific correlations localized in component A (denoted by green color of the nodes), covering primary metabolism and most amino acid synthesis pathways. Nevertheless, even in this tightly connected subnetwork, heat hubs, representing the peak of heat stress connectivity, may be discriminated from control and lactose shift hubs. Lactose shift and control conditions on the other hand seem to overlap to some extent in range of component A.

The control conditions contains relatively few new connections in comparison to the stable component network, highlighting mainly primary metabolites of component A as network hubs. Importantly under control conditions metabolites of component B form a clear separate community.

The lactose shift derived network on the other hand contains a number of metabolites as network hubs which are represented as isolated nodes without any connections in the stable component network, such as PEP, leucine, asparagine or glycerol-3-phosphate. In this regard the lactose shift network seems to be very distinct from all the other conditional networks, which indicates highly specific change in the metabolic system.

Oxidative stress hubs are localized mainly in component A, however among these we also observe several metabolites belonging to other components. Uracil, which represents an isolated node in the stable component network, exhibits several oxidative stress specific negative correlations with metabolites of component A. This feature is shared by cold and oxidative stress networks and is in contrast to heat stress and control conditions, where uracil displays a positive correlation.

The most sparse pattern of correlations is exhibited by the cold stress network. Similarly to lactose shift, cold stress engages numerous metabolites without connectivity in the stable network, such as leucine, oxalate, trehalose, phenylalanine or uracil. The first two are network hubs, next to methionine, ethanolamine, 2-hydrohyglutarate and several not precisely annotated compounds. In the cold stress specific network metabolites of component B form a partially separated community, which makes it similar to control and lactose shift. In addition to being present in part of the connectivity within component A and B, a significant number of the cold stress-dependent correlations are localized peripherally to the other stresses, indicating a significantly different way of stress adaptation.

Heat stress finally exhibits a compact connectivity enrichment mostly due to a significant increase in connectivity between members of component A e.g. alanine, threonine or fumarate. Phenylalanine, trehalose, uracil and arginine, which are isolated nodes in the stable network component become heat specific hubs.

The analysis of the networks resulting from the various treatments thus clearly shows a high degree of specificity. As outlined in the Introduction one of our goals was to assess the possibility to use a network based analysis for the identification of metabolites which might be crucial for one or several stress conditions. Hubs which manifest under one or more stress conditions might represent metabolites important for adjusting the cellular machinery towards the new environmental condition and thus could represent candidates for metabolic adjustment and/or signaling during this phase. We will next discuss four examples in an exemplary fashion in some detail in order to introduce the concept before a more comprehensive analysis.

With respect to using the conditional hub property as an identified for an interesting metabolite three situations can be distinguished: Metabolites which present a hub only for one specific condition, for more than one or for all stress conditions.

**Figure 5:**
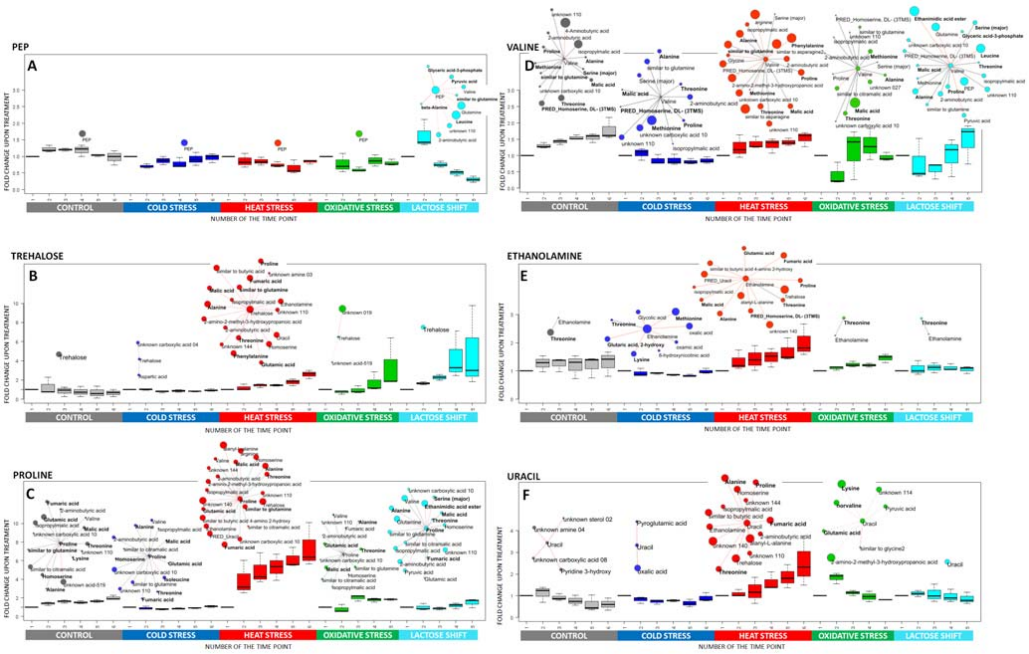
Relative changes of metabolites concentrations and changes in their network connectivity. Pattern of concentration changes for six selected metabolites (A: PEP (phosphoenolpyruvate); B: trehalose; C: proline; D: valine; E: ethanolamine; F: uracil) as a function of time during different stress and control conditions ( the time points relate to the time points as indicated in figure 1a) and part of the respective metabolite-metabolite correlation network observed in response to applied treatments showing the neighbours of these six metabolites ( if any). Boxplots are generated using fold change values of individual biological replicates in relation to the average value in time point 1 – before the treatment. Significantly correlating metabolites are displayed as nodes connected to the metabolite of interest with an edge. Correlation analysis confers only time points 2,3,4,5 as described in the main text. Red edge - treatment specific correlation; gray edge - correlations common for all treatments; solid edge – positive correlation; dashed edge – negative correlation. The size of the node is proportional to the number of stress specific correlations exhibited by this metabolite. Metabolites exhibiting significant changes in absolute concentrations are marked with a bold font face.

An example for the first group is PEP. It is an isolated node in all treatments, except the lactose shift, where it becomes one of the main network connectors (Figure 5a.). In glucose starvation, induced by lactose diauxie, rapidly increasing phosphoenolopyruvate (PEP) shows especially high number of negative correlations with several transiently decreasing amino acids, such as valine (Figure 5c.), glutamine, leucine or homoserine. PEP is a phosphate donor for phosphotransferase system (PTS) responsible for glucose import and its accumulation has been recently proposed to be a direct effect of low glucose import from glucose deficient medium [31]. This interpretation is supported by observation that PEP level decrease to the initial level after adaptation phase (40 min, data not shown). Interestingly, beside several amino acids PEP correlates negatively with pyruvate and malate. This indicates that probably accumulation of PEP is a result of the shift of metabolic resources coming from other parts of central metabolism. This might also result in decreased pools of some amino acids, especially if an increased protein synthesis occurs. Such interpretation, although speculative and not directly supported by measuring of metabolic fluxes, might be taken as an example of hypothesis creation based on analysis of condition specific metabolite-metabolite correlations. We would like to stress that as shown in figure 5a PEP is detected under all conditions and actually based on changes in concentration alone, i.e. the classical approach would not be detected as a putative important metabolite as shown in figure 5a. However by performing the network analysis it clearly comes out as a major hub suggestive of an important role during lactose diauxic shift which actually is in agreement with previously published findings [31].

A further example is trehalose which displays a hub property under heat conditions thus suggesting its importance for adaptation to heat stress (figure 5b). Trehalose indeed has been reported as a stress related compound, as a well-known storage compound and protectant against various stress conditions. It was shown in yeast and *E. coli* that trehalose is accumulated under stresses, such as: heat and cold [53], [54], osmotic stress [55], or oxidative stress [56]. The actual molecular role of trehalose has not been unambiguously ascertained but there are suggestions that it could increase general protein stability [57] and take part in stabilization of the cell membrane [53] which rather has been discussed in case of heat stress and not so much for oxidative stress. This would indeed be in nice agreement with the network based identification which identifies trehalose as an important hub in heat stress and much less so for cold and oxidative stress (Figure 5e).

Yet another interesting compound connected with heat stress and highlighted by correlation analysis, is proline (figure 5c). A close and stable connection with glutamate might be related to its biosynthesis pathway, however a number of conditional connections indicates involvement of proline in various responses of the cell to exogenous stimuli. In case of heat stress proline is located in the same densely connected network region as trehalose and ethanolamine and thus connects most of their neighbors too. Similar to trehalose, proline has been reported to enhance stability of the proteins in freezing [58] and as an important compound in membrane stabilization during freezing [59], dehydratation [60] and elevated temperatures [61]. It has been demonstrated also that the simple addition of proline to growth media had a protective role against heat and cold in *E. coli* [62].

The metabolites described above were selected because of their conditional hub property. As a contrasting example we show the network property of valine (figure 5d), which has a defined stable connectivity (12 stable neighbors) and just several stress specific correlations, despite of substantially different patterns of its accumulation.

The examples described above were based on identifying metabolites which display a specific network property (hub) under one or few stress conditions. We decidedly discussed three examples (PEP, trehalose and proline) where there is substantial evidence in the scientific literature for their importance for stress adjustment thus strengthening our concept. Figure 5 f and g show two more examples where metabolites based on a conditional hub property are identified, i.e. ethanolamine and uracil. Specifically the case of ethanolamine is interesting and shall thus be discussed again in some more detail. It displays a stable correlation with threonine across all the conditions however more important shows a substantial increase in connectivity under cold and heat stress. Again it is important to note that changes in ethanolamine concentration alone would not result in its immediate identification again demonstrating the potential of correlation analysis for identification of important metabolites. Ethanolamine is the second most abundant head group for phospholipids in biological membranes; however, the biophysical properties of phospholipids are mainly defined by the fatty acid component [62]. The specific detection of ethanolamine under both cold and heat stress may suggest a link between lipid metabolism and temperature stress. This is supported by observation that correlations in frame of module C, conferring such lipid related compounds as heptadecanoate, palmitoleate and palmitic acid are highest in case of cold stress. It was shown in various biological models that a decrease in temperature causes changes in membrane lipid composition [63], [64], [65]. Finally during longer periods of unfavoured temperatures the membrane lipid composition of *E. coli* can change toward an increase of more saturated fatty acids under heat [64] and unsaturated fatty acids under low temperature [66] again lending support to the identification of ethanolamine as an important metabolite involved in the adjustment of the system towards the two temperature stresses.

Table 6 finally gives an overview of all metabolites being a conditional hub under one or more stress conditions. Furthermore the reverse may also be interesting, i.e. a metabolite losing its hub property under one specific stress conditions. These might be traced in supplementary Table S1.

**Table 6:** Stress specific hubs.

Control	Heat stress	Cold stress	Lactose shift	Oxdidative stress	Cold/Heat stress	Common stress response
Pyruvate	Trehalose	Ethanolamine	ethanimidic acid	Lys	Ethanolamine	malate
Norvaline	Ala	Met	Leu	Pyroglutamate	Thr	Val
isopropyl-malate	Uracil	oxalate	glycerol-3-phosphate	Norvaline	Phe	Ala
4-hydroxy-benzoate	Phe	N-carbamyl-Asn	PEP	Cys	2-aminobutyrate	Pro
Norleucine	Arg		Glu	Malate		homoserine
Gln	Pro		2-ketobutyrate	Asn		2-aminobutyrate
Thr	Ethanolamine		Succinate	Glu		
				Putrescine		

Stress specific hubs and chosen metabolites being hubs in cold/heat stress and in common stress response. Only identified metabolites are mentioned, the full table available in supplementary materials.

### Conclusion

We here describe results obtained by following the response of *E. coli* towards four different stress conditions and control conditions over time on the metabolite level. We construct metabolic networks based on metabolite –metabolite correlations and identify various network topology parameters. We show that the network differs considerably between the five different conditions. A stable component of the network containing those correlations found under all conditions is shown to reflect biochemical pathways as described e.g. in EcoCyc. We demonstrate that network properties displayed by metabolites under only one or several stress conditions can be used as a novel filter to identify metabolites which very likely play an important role during the adjustment phase of E. coli to the specific prevailing condition. As this novel approach does not rely on major changes in concentration to identify metabolites important for stress adaptation, but rather on the changes in network properties with respect to metabolites, we believe this to be a useful complementary technique in addition to more classical approaches.

## Materials and Methods

### Bacterial strains and Growth Conditions

We used wild type *Escherichia coli* K12 MG1655 strain, obtained from the American Type Culture Collection (ATCC® 700926). Bacteria were grown from the single colony aerobically in 1000 ml flasks containing MOPS minimal medium [67] (obtained from Teknova CA, product number M2006) in 37°C. All cultures were grown aerobically in a thermostatically controlled 37°C culture room, with aeration provided by magnetic stirrer. The temperature and pH were carefully monitored during growth. Starting cultures were inoculated from a single colony and grown overnight. Each experimental culture was then inoculated from such an overnight culture at a dilution of 1:20 into 150 ml fresh MOPS minimal medium in a 1000 ml flask. Growth of cultures was monitored by measuring optical density (OD) at 600 nm using an Eppendorf Biophotometer. All cultures were grown until early-mid log phase (OD 0.6), at which point each of the perturbations was applied.

Oxidative stress: 200 µg/ml of 30% pre-warmed hydrogen peroxide (Fluka) was added to constantly stirring cultures. The amount of hydrogen peroxide used for the stress was calculated to cause a non-lethal ∼40 minute lag phase. This was monitored by plating on solid LB medium and calculating viable cell number.

Cold stress: Cultures were transferred from 37°C into an ice cold water bath in order to lower the temperature, while stirring, to 16°C in less than 2 minutes. When 16°C had been attained, flasks were transferred to a 16°C water bath while constantly stirring.

Heat stress: Cultures were transferred from 37°C to a 50°C water bath. While stirring, the temperature of each culture was raised to 45°C in under two minutes. The constantly stirring cultures were then transferred to a 45°C water bath to maintain this temperature. In both temperature treatments the temperature was constantly monitored ensuring that the cold treatment culture never exceeded 16°C and the heat treatment culture never dropped below 45°C

Glucose–lactose shift: Carbon source concentrations of 0.15% lactose and 0.05% glucose were used. This meant that the adaptation phase was observed at OD∼0.6.

### Sampling

The first two time points were taken before stress at OD 0.5 and 0.6. Following stress application the subsequent sampling time points were at 10 minute intervals for up to 40 minutes (lactose shift and oxidative stress) or 50 minutes (cold, heat and control). Rapid filtering as described by Bolten et al using 2.5 cm diameter, 0.45 µm pore size Durapore® filter disks (Millipore Corporation, MA) and a vacuum manifold and pump was used. Metabolite (1 ml) and transcript (4 ml) samples were taken simultaneously. Filters with adhering bacteria were rapidly transferred into 2 ml centrifuge tubes and flash frozen in liquid nitrogen. The whole process took less than five seconds (metabolites) or 10 seconds (transcripts) per sample from sampling to flash freezing in liquid nitrogen.

For GC-MS metabolite analysis, each of the filter discs with adhered bacteria was extracted in 500 µl cold Methanol (Merck) as this has previously been shown to be superior to hot methanol, hot ethanol, cold perchloric acid, hot alkaline and cold methanol/chloroform extraction protocols [68]. The extraction solution contained 0.1 µg/ml cholesterol as an analytical internal standard. Tubes were subsequently shaken at 4°C for 10 min at 1000 rpm and again frozen in liquid nitrogen. This freeze-thaw cycle was repeated to ensure cell membrane rupture. Finally filters were removed, samples centrifuged for 3 min at 14,000 rpm at 4°C (Eppendorf 5417R) and 450 µl of the supernatant transferred into new 2 ml centrifuge tubes. These samples were then dried to complete dryness in a rotary vacuum centrifuge device. Dried samples were subsequently stored at −20°C for a maximum of two weeks before analysis.

### Extraction and Measurement of Metabolites

Derivatization of the samples was done following the modified version of protocol described in [69] including methoximation, through a 90 min 30°C reaction with 5 µl of 40 mg/ml methoxyamine hydrochloride (Sigma-Aldrich) in pyridine (Merck), followed by derivatization of acidic protons via a 30 min 37°C reaction with the addition of 45 µl MSTFA (N-methyl-N-trimethylsilyltrifluoroacetamide) (Machery-Nagel). 1 µl of the derivatized sample was injected onto the column and analysis was commenced in non-split mode.

GC-MS hardware comprised an Agilent 6890 series GC system fitted with a 7683 series autosampler injector (Agilent Technologies GmbH, Waldbronn, Germany) coupled to a Leco Pegasus 2 time-of-flight mass spectrometer (LECO, St. Joseph, MI, USA). Identical chromatogram acquisition parameters were used as those previously described (Weckwerth et al, 2004). Chromatograms were processed using Leco ChromaTOF software (version 3.25) and analytical peaks determined using the method of Lisec et al. 2006 [22] with a modified peak picking algorithm which searches for local apex intensity from all mass traces in raw chromatograms. All chromatograms were next manually checked. The identification of metabolites was confirmed by comparing spectrum and retention index of given peak with chemical library standards. Also to exclude possible export errors from Leco ChromaTOF software to cdf file format the height of given peak was compared with row chromatographic data. Peaks were also curated according to their quality; co-eluting, overloaded and too small (height<100) peaks were excluded from the analysis. All data were normalized to cell number and the chromatographic internal standard.

### Metabolic Data Analysis

Each experimental condition was independently repeated three times and in each of these repetitions, three technical replicas were made. The row data was normalized to optical density of the sample (number of cells) and peak intensity of internal standard (cholesterol).

### Correlation Analysis

Data used for correlation analysis cover time points from stress application to the beginning of stationary phase and include 3 biological replicas per time point. Each biological replica comes from 3 technical ones. Because of different dynamics of growth upon stress the data includes different number of time points for different stresses: 5 time points for control, heat and cold treatments and 4 time points for oxidative stress and lactose shift. For this data we used non-parametric Spearman's rank-order correlation to obtain correlation matrix for each of the investigated datasets. To reconstruct individual networks the correlation coefficient matrices were transformed into binary adjacency matrices according to a<0.01 [99%] p-value threshold corrected for a multiple comparisons using Bonferroni correction. To diminish the influence of single or few measurements on the observed correlation coefficient, all the significant correlations were recalculated using non-parametric bootstrap analysis using 1000 bootstrap samples.

### Stable Component

To find the significant correlations, which are also conserved in all of the networks we use a simple two step approach.

1. In the first step a “union” network is reconstructed. This is equivalent to superimposing the networks from specific conditions one on the other. Thus an edge in the resulting network exists if the underlying metabolite-metabolite correlation is found significant at least in one of the treatments. Edges of the union network gains a weight from 1 to 5, in respect to the number of conditions in which they were found, however in our study all the edges of union network were treated equally.
2. In the second step a correlation homogeneity test is performed for all edges of the union network. For each edge of the union network 5 correlations coefficients coming from 5 individual stress networks are taken. After transformation into their Z-scores, they are tested for homogeneity of the distribution. In result only those edges, which pass a Chi square test with p-value cutoff <1E-4 are taken as stable. For a proper homogeneity test performance all the correlations, which pass the absolute significance threshold, are treated equally.

Each edge of the resulting “stable network” (“stable component” as referred in the text) passes two criteria – it is significant at least in one of the individual networks and the distribution of correlation coefficients exhibit a homogeneous distribution for all of the individual networks. Accordingly we refer to the edges of a stable component as to “stable edges” or “stable connections” and to the rest as to “conditional” or “condition/stress specific”.

### Network Topology Analysis

Modularity analysis was performed using Newman's bottom-up centrality, random walk and leading eigenvector algorithms. Topology analysis was performed using cumulative degree distribution and non-linear least squares for distribution function parameters estimation. Akaike Information Criteria (AIC) for goodness-of-fit determination was used to estimate the optimal fit [36], [37]. Shortest path analysis used for network comparison was performed to compare the differences in distance for each pair of metabolites, when different conditions are applied to the system. For this purpose, geodesic matrices were obtained for each network and normalized to a network diameter. Subsequently for each network-to-network comparison path length difference matrix was computed. The obtained result was compared to 1000 random network rewirings preserving the original degree distribution. An average path length distribution for random networks was used to estimate comparison significance.

### EcoCyc Network Reconstruction

Database of the metabolic reactions was downloaded from EcoCyc [http://www.ecocyc.org]. Initially 2-mode network was reconstructed, containing metabolites and reactions as nodes and edges representing contribution of metabolites in particular reactions. Subsequently the currency metabolites as main connectors, represented by ions, cofactors and small inorganic molecules were removed from the network. The choice of them was reasoned by a number of nonspecific network shortcuts they introduce into the metabolic network structure. Among removed metabolites were: H2O, ATP, ADP, Pi, PPi, NAD, NADH, CO2, H+, NH4+, NADP, NADPH, CO-A, AMP, O2, CMP, GTP, GDP, UDP, CTP, UMP, HCO3, SO3, UTP, TMP, H2O2. The list was composed basing on previous published studies [45], [46], [47]. Many non-defined molecules were also removed, including such as “polypeptide” or “disaccharide”. Resulting network was transformed into classical 1-mode view, where nodes representing metabolites are linked by edges representing reactions. A main connected component of the network consists of 613 metabolites connected by 568 reactions.

### Software

Network properties analysis and all operations were performed using R 2.7.1 software [70] and Pajek 1.22 [71]. Base set of R packages was used for statistical analysis of the metabolic data, igraph 0.5.2 R package [72] was used for graph analysis and operations on the networks. Visualization and feature mapping was done using Cytoscape 2.5.1 software [73].

## Supporting Information

Figure S1 E. coli growth curves. Growth curve ( optical density) of unperturbed ( left) and perturbed ( right; oxidative stress experiment is shown as an example) E. coli cultures. The x-axis displays the growth time in minutes. The numbers next to the curve refer to the numbering used in Figure 1b and supplementary Figure 1.
(0.08 MB TIF)

Figure S2 Comparison of metabolic time courses observed in different conditions. K means clustering of metabolite behavior in control and stressed cultures. Changes observed in metabolites during early and mid logarithmic growth phase (time points 1 to 6) and stationary phase (time point 8 to 10) for control culture. Time points 1 to 10 represents unequal time spans after application of a stress factor. These are respectively: 0, 10 min, 20 min, 30 min, 40 min, 90 min, 150 min, 210 min, 235 min, 260 min. The growth curve corresponding to the time points indicated is shown in Figure 1a. 10 clusters for each condition were identified. Time courses of all metabolites for particular conditions are located in rows. Colors representing clusters identified for particular conditions are located in columns. Value denoted on the plot represents Mantel statistics score, comparing euclidean distance matrices obtained for the corresponding treatments. For each pair significance of the test statistics was approaching 0.
(0.09 MB TIF)

Table S1 Basic information about metabolites analyzed in this study. It concerns: metabolites quantification, their variance of its concentration in different conditions, reference to significant accumulation or degradation, basic network properties and KEGG identifiers.
(0.10 MB XLS)

Table S2 Pearson correlation coefficient matrices used for the analysis.
(1.90 MB XLS)
