# Supplementary figures and images for: Stability of Metabolic Correlations under Changing Environmental Conditions in Escherichia coli – A Systems Approach
Source: PLoS One. 2009 Oct 15;4(10):e7441. doi: 10.1371/journal.pone.0007441 (PMC2759078; doi:10.1371/journal.pone.0007441)

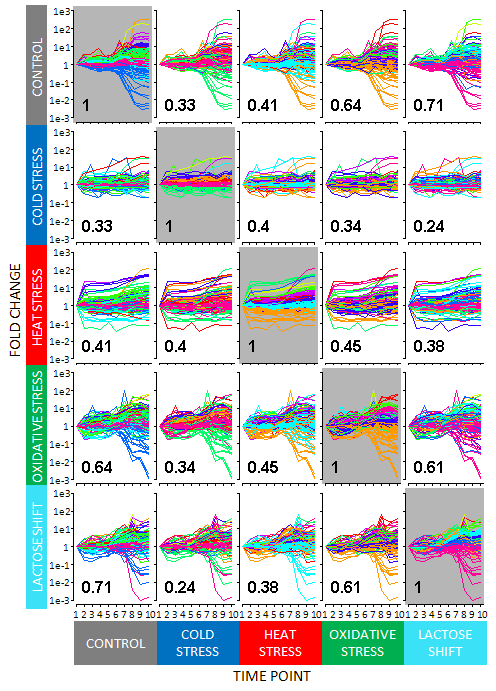

Supplement: Figure S1 — E. coli growth curves. Growth curve ( optical density) of unperturbed ( left) and perturbed ( right; oxidative stress experiment is shown as an example) E. coli cultures. The x-axis displays the growth time in minutes. The numbers next to the curve refer to the numbering used in Figure 1b and supplementary Figure 1. (0.08 MB TIF) [file pone.0007441.s001.tif]

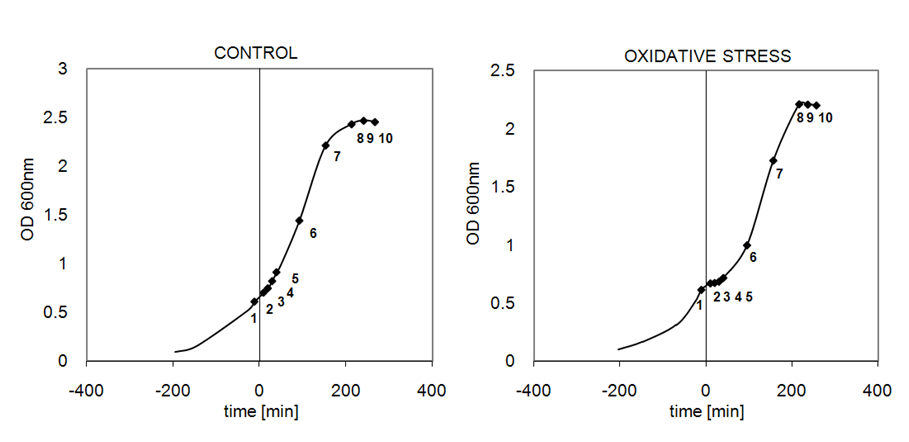

Supplement: Figure S2 — Comparison of metabolic time courses observed in different conditions. K means clustering of metabolite behavior in control and stressed cultures. Changes observed in metabolites during early and mid logarithmic growth phase (time points 1 to 6) and stationary phase (time point 8 to 10) for control culture. Time points 1 to 10 represents unequal time spans after application of a stress factor. These are respectively: 0, 10 min, 20 min, 30 min, 40 min, 90 min, 150 min, 210 min, 235 min, 260 min. The growth curve corresponding to the time points indicated is shown in Figure 1a. 10 clusters for each condition were identified. Time courses of all metabolites for particular conditions are located in rows. Colors representing clusters identified for particular conditions are located in columns. Value denoted on the plot represents Mantel statistics score, comparing euclidean distance matrices obtained for the corresponding treatments. For each pair significance of the test statistics was approaching 0. (0.09 MB TIF) [file pone.0007441.s002.tif]
